# Electrical Resistance Reduction Induced with CO_2_ Laser Single Line Scan of Polyimide

**DOI:** 10.3390/mi12030227

**Published:** 2021-02-24

**Authors:** Zhongke Wang, Kok Keat Tan, Yee Cheong Lam

**Affiliations:** 1 SIMTech-NTU Joint Laboratory (Precision Machining), Nanyang Technological University, 50 Nanyang Avenue, Singapore 639798, Singapore; 2 Singapore Institute of Manufacturing Technology (SIMTech), A*STAR, 2 Fusionopolis Way, Singapore 138634, Singapore; tank0233@e.ntu.edu.sg; 3 School of Mechanical and Aerospace Engineering, Nanyang Technological University, 50 Nanyang Avenue, Singapore 639798, Singapore

**Keywords:** CO_2_ laser irradiation, polyimide film, graphene structure, electrical conductivity, MicroRaman spectra

## Abstract

We conducted a laser parameter study on CO_2_ laser induced electrical conductivity on a polyimide film. The induced electrical conductivity was found to occur dominantly at the center of the scanning line instead of uniformly across the whole line width. MicroRaman examination revealed that the conductivity was mainly a result of the multi-layers (4–5) of graphene structure induced at the laser irradiation line center. The graphene morphology at the line center appeared as thin wall porous structures together with nano level fiber structures. With sufficient energy dose per unit length and laser power, this surface modification for electrical conductivity was independent of laser pulse frequency but was instead determined by the average laser power. High electrical conductivity could be achieved by a single scan of laser beam at a sufficiently high-power level. To achieve high conductivity, it was not efficient nor effective to utilize a laser at low power but compensating it with a slower scanning speed or having multiple scans. The electrical resistance over a 10 mm scanned length decreased significantly from a few hundred Ohms to 30 Ohms when energy dose per unit length increased from 0.16 J/mm to 1.0 J/mm, i.e., the laser power increased from 5.0 W to 24 W with corresponding power density of 3.44 × 10 W/cm^2^ to 16.54 W/cm^2^ respectively at a speed of 12.5 mm/s for a single pass scan. In contrast, power below 5 W at speeds exceeding 22.5 mm/s resulted in a non-conductive open loop.

## 1. Introduction

Laser ablation of polymers has been studied since the early 1980s [1]. Ultraviolet laser ablation of polyimide films in air is a multiphoton excitation process resulting in polyimide photochemical decomposition into oxides of carbon and elemental carbon, etc. [2,3]. Electrical conductivity in polyimide is induced through the release of nitrogen and oxygen to result in graphitizing carbon sheets [4]. The electrical conductivity of polyimide can increase up to 15~16 orders of magnitude under the irradiation of ultraviolet laser [5]. Recently, graphene stacked structures in polyimide film was observed under CO_2_ laser irradiation [6]. Since then, laser-inducing graphene on polyimide has attracted much attention [7]. Research on graphene has led to its successful applications from electronics to catalysis due to the excellent chemical and electrical properties of graphene [8,9]. It is well known that polyimide is an important material for microelectronics. The combination of graphene and polyimide has made polyimide film even more fascinating for applications in microelectronics and sensing, such as supercapacitor energy storage devices [7,10] and stretchable sensors [7,11].

Nonetheless, for practical application, the laser irradiation parameters have to be well understood for the generation of graphene structure in polyimide surface to achieve induced conductivity. In addition to laser power and scanning speed, the effect of laser beam scanning passes, pulse frequency, etc. on graphene synthesis and the layers of graphene structures formed are yet to be investigated appropriately. Furthermore, the electrical resistance of an individual line scanned on the polyimide surface is critical for practical manufacturing of electrical circuits in polyimide. Thus, this study aims to explore the key laser parameters to produce graphene structure on the polyimide film as the graphene structure determines the electrical property of laser modified polyimide film. More importantly, this investigation attempts to understand the morphology of the graphene structure on the polyimide film surface induced by irradiation with various CO_2_ laser parameters. The interplay of the various key processing parameters is to be revealed for the determination of the optimum parameters for an effective and efficient enhancement of electrical conductivity.

## 2. Experimental

The polyimide film employed in this investigation was a commercially available 75 μm thick polyimide (Kapton) foil. The CO_2_ laser used was Synrad Fire-star Ti60, which had a wavelength of 10.64 µm. The laser has a full power of 60 W, which was able to modulate at different pulse repetition rates ranging from 1 kHz to 100 kHz. The laser beam was focused through a galvo scanner focal lens with a focal length of 160 mm and a focal diameter of about 430 µm. Laser parameters investigated were power level, scanning speed, pulse frequency and number of passes. Power levels of 5% to 50% were used with the scanning speed varied from 5 mm/s up to 250 mm/s, and pass number of one up to ten.

During laser ablation, polyimide film was exposed to air. After laser modification, a layer of black compound could be observed along the scanned line on the surface of the film. The black scanning line was then tested with resistivity meter probes (34401A 6½Digit Multimeter Agilent) placed over two ends of a 10 mm length of the scanned line. The electrical resistance over the irradiated 10 mm line could then be quantitatively measured. The surface morphology of irradiated polyimide was characterized with a Scanning Electron Microscope (SEM/EDS) from Joel JSM-IT300LV, Oxford XMax80.

Raman spectroscopy with a laser source of 785 nm and 50× objective magnification was used to determine the chemical make-up of the irradiated line. The Raman Imaging Microscope was from inVia Raman Microscope, Renishaw. Raman laser power used for analysis should be low so as not to modify the surface, but high enough to provide a good signal to noise ratio. For most samples, a laser power of 5% was used to obtain clear peak profiles. A lower power of 1% was used instead for samples which were poorly conductive/non-conductive and produced too much fluorescence at 5% laser power. The Raman spectrum scanned was from 800 shift/cm^−1^ to 3000 shift/cm^−1^.

## 3. Results and Discussion

### 3.1. Electrical Resistance of the Laser Irradiation Line

**Figure 1:**
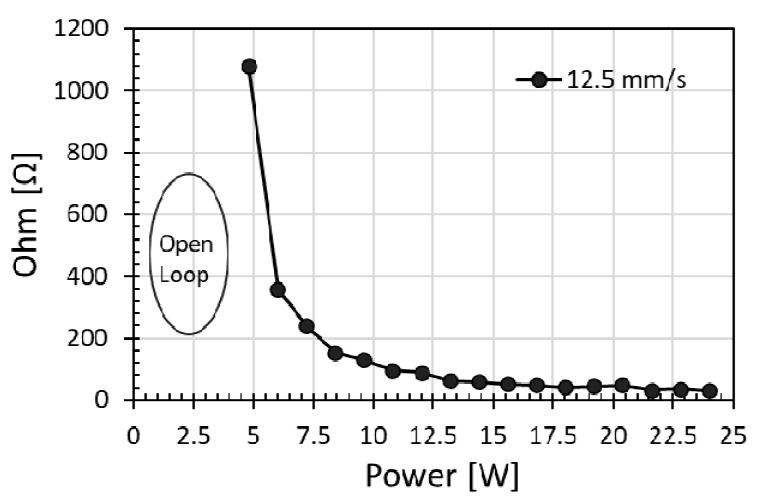
Electrical resistance decreased with increasing laser power with a single pass scanning at a scanning speed of 12.5 mm/s and power modulation repetition rate of 90 kHz.

To avoid excessive charring of polyimide, investigation was first carried out with low laser power, and subsequently the power was increased. Figure 1 shows that the electrical resistance obtained with a single laser scan at a speed of 12.5 mm/s. For laser power less than approximately 5 W, the polyimide surface was not modified for a noticeable decrease in resistance, and a nonconductive open loop was obtained. When the laser power was increased from 5.0 W to 24 W with corresponding power density of 3.44 × 10 W/cm^2^ to 16.54 W/cm^2^ respectively, the measure electrical resistance decreased from a few hundred Ohms to 30 Ohms over the irradiated 10 mm length, indicating the significant decrease of resistance with an increase of laser power. In contrast, non-conductive open loop was produced with irradiation power below 5 W at speeds exceeding 22.5 mm/s. Thus, to achieve low resistance, i.e., high electrical conductivity, sufficiently high laser power was recommended to irradiate the polyimide surface.

**Figure 2:**
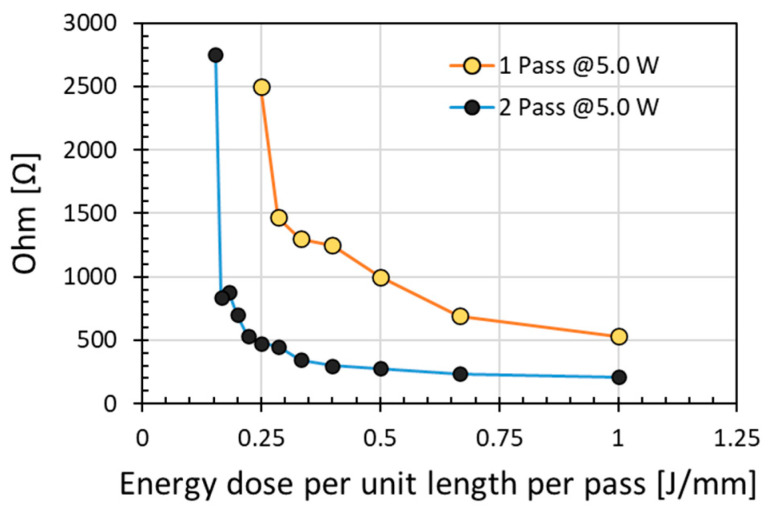
Electrical resistance increased with energy dose per unit length applied to irradiate polyimide surface (power of 5.0 W with corresponding power density of 3.44 W/cm^2^).

During laser irradiation, resistance modification was a function of both the irradiation laser power level and the irradiation time. Indeed, the modification would depend on the laser energy dose available, namely energy density (J/cm^2^), for absorption by the material. For a constant laser spot area, the combined effect of laser power and scanning speed may be conveniently discussed with energy dose per unit length expressed as power per unit scanning speed ((W/(mm/s) = (J/mm)). Thus, instead of plotting the changes of electrical resistance with either power or scanning speed, the resistance values could be plotted as a function of energy dose per unit length as shown in Figure 2. It depicts the electrical resistance values obtained at various speeds from 5 mm/s to 35 mm/s with either 1 or 2 passes scanning at a constant laser power of 5.0 W (power density of of 3.44 W/cm^2^). Clearly, lower resistance was produced with increasing energy dose per unit length from 0.154 J/mm at speed of 35 mm/s to 1.0 J/mm at speed of 5 mm/s. As shown in Figure 2, double the energy dose per unit length by scanning twice could significantly induce further reduction of the electrical resistance.

Reducing the scanning speed was not effective in compensating the decreasing laser power to result in low electrical resistance. This clearly indicates that a certain laser power density was required to induce electrical resistance reduction. Scanning at the very slow speed of 5 mm/s, the electrical resistance remained above 200 Ohms despite the sample was scanned twice. Open loop resistance was measured at speed higher than 35 mm/s. To achieve low electrical resistance, these results indicate that it is not efficient nor effective to utilize a laser at low power but compensating it with a slower scanning speed or having multiple scans. Sufficiently high laser power level, thus power density, was preferred for low electrical resistance.

**Figure 3:**
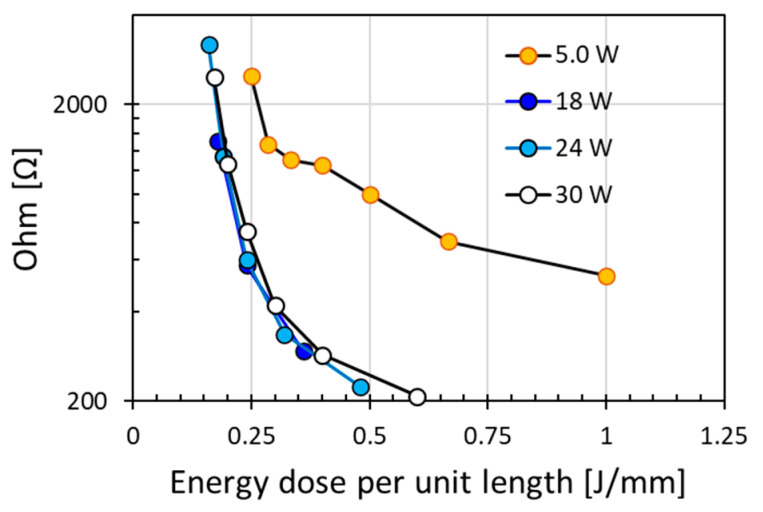
Electrical resistance increased with energy dose per unit length applied to irradiate polyimide surface for a single pass scanning at various laser powers (5, 18, 24, and 30 W gave 3.44, 12.40, 16.54, and 20.67 W/cm^2^, respectively).

Although high laser power is desirable for achieving low electrical resistance, scanning speed is an important parameter for the final outcomes. The combined effect of power and speed may be reflected conveniently by the energy dose per unit length applied. A similar energy dose per unit length value could be obtained by using either high power with high speed or low power with low speed. For example, a high energy dose per unit length could be obtained by increasing laser power or decreasing scanning speed. Except for the low laser power of 5.0 W, Figure 3 shows that similar value of modified resistance was obtained with similar energy doses per unit length even if the power level or density was different, with the electrical resistance significantly reduced with increasing energy dose per unit length. However, when the laser power is too low, i.e., at laser power of 5.0 W, the resistance obtained was much higher than that for higher laser power for the same energy dose per unit length. This might be attributed to the effective width of laser irradiation. The low laser power of 5.0 W produced a narrow modified scanning line width due to the low pulse energy, as compared to the wider line width produced by higher laser power with high pulse energy. Above a certain power level or power density, the increase in modified line width would be much less sensitive to the increase in power density. This modified line width has a direct effect on electrical resistance as it would be a function of the physical size of the modified resistive/conductive line. Further discussion on the irradiation line width can be found in later section.

Figure 3 depicts that the results obtained at energy doses per unit length of 0.16–1.0 J/mm with scanning speeds of 5 to 174 mm/s under various laser powers. Indeed, the reduction of resistance plateau to a low and similar value for the three moderately high laser powers investigated. These results indicate the interplay between laser power and scanning speed for achieving low resistance. By contrasting the results in Figure 3 to that in Figure 2, it can be deduced that a moderately but sufficiently high laser power is far more efficient and effective than a low laser power. At the low laser power of 5.0 W, Figure 2 indicates that much higher resistance was obtained at a significantly lower scanning speed, even for 2 pass scanning. These analyses indicate that a moderately slow speed and a moderate laser power were optimum for modifying the polyimide surface from an electrical insulator to an electrical conductor.

**Figure 4:**
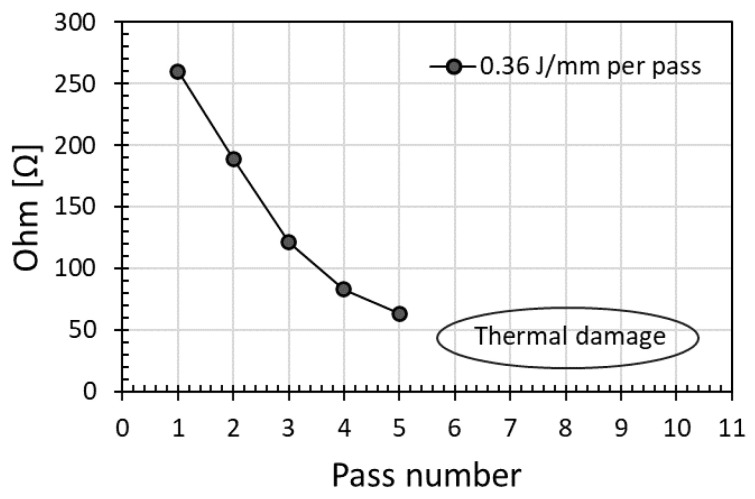
Effect of scanning pass number on electrical resistance at a laser power of 18 W and a scanning speed of 50 mm/s (which produced an energy dose per unit length of 0.36 J/mm per pass).

The increase of energy dose per unit length by increasing the number of passes could be different from that by increasing the laser power, as pulse energy or power density was high at high laser power. As indicated in Figure 2, at low laser power, 2 pass scanning had resulted in lower electrical resistance than a single pass scanning. As such, intuitively, multiple scans could be utilized similarly at higher laser power to further reduce the electrical resistance. Indeed, as indicated in Figure 4, electrical resistance decreased with an increase in energy dose per unit length induced by increasing pass number. Unfortunately, when scanning exceeded pass number 5 (i.e., energy dose per unit length exceeded 5 × 0.36 J/mm), instead of surface modification along the scanning line, it caused thermal burning damage instead, see Figure 4; the electrical resistance could not be properly measured as the scanning line did not appear as a line anymore due to the damage. This indicates that the effect of an increase in energy dose per unit length by increasing the number of passes would be different from that by increasing the laser power, as pulse energy or power density was high at high laser power. For comparison, the lowest resistance of 63 Ω obtained by multiple scans in Figure 4 was still higher than the 30 Ω obtained with a single pass scan in Figure 1 albeit at a higher laser power at a slower scanning speed; these results indicate that multiple scanning was not an efficient way to achieve a low electrical resistance. The initial modified layer could block the subsequent laser beam to further interaction with polyimide, with less effective modification beyond the previously modified layer as the pass number increased. Instead, additional scanning might cause damage of the previously modified layer.

It will be of interest to investigate the effect of pulse repetition rate on electrical resistance. The range of pulse repetition rate investigated was from 1 kHz to 100 kHz, which was the range of the laser system. In general, for a laser beam at a given laser power, the pulse energy decreases with increasing pulse frequency.

**Figure 5:**
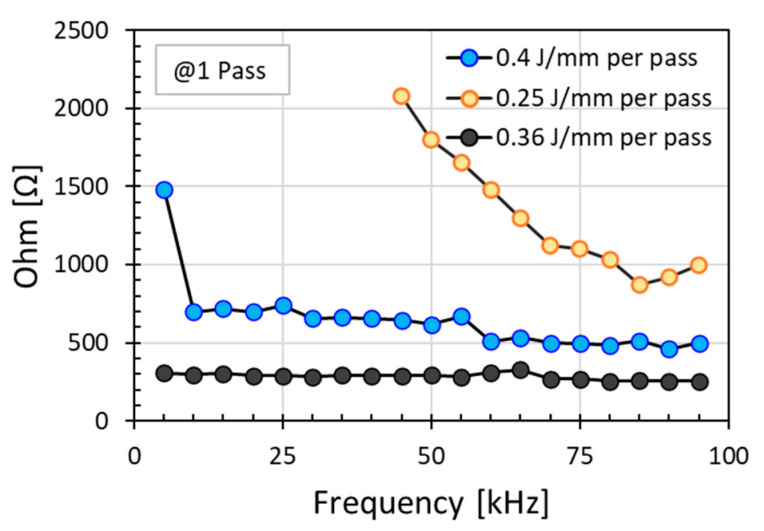
Effect of pulse frequency on electrical resistance under irradiation at different energy dose per unit length in a single scan (0.4 J/mm produced from 5.0 W and 12.5 mm/s, 0.25 J/mm from 5.0 W, and 20 mm/s; and 0.36 J/mm from 18 W and 50 mm/s).

Figure 5 presents the effects of pulse frequency on the resistance measured at different energy doses per unit length. These energy doses per unit length were obtained with different combination of power and scanning speed, namely energy dose per unit length of 0.4 J/mm obtained with power of 5.0 W and speed of 12.5 mm/s, energy dose per unit length of 0.25 J/mm with 5.0 W and 20 mm/s, and energy dose per unit length of 0.36 J/mm with 18 W and 50 mm/s. Intuitively, one would expect that higher energy dose per unit length should produce lower electrical resistance. However, Figure 5 indicates that higher energy dose per unit length of 0.4 J/mm produced higher resistance values (blue curve) than lower energy dose per unit length of 0.36 J/mm (black curve). This can be attributed to the pulse energy or the power density which was higher at high laser power (18 W) for the lower energy dose per unit length of 0.36 J/mm as compared to that at low laser power (5 W) for the higher energy dose per unit length of 0.4 J/mm. This indicates that pulse energy plays a critical role as well in determining the modified resistance in addition to the total amount of the energy (expressed as energy dose per unit length) deposited into polyimide.

At the lower power of 5.0 W, the pulse energy was low, the electrical resistance decreased with increasing pulse frequency; this decrease of resistance with frequency was much more significant at the high scanning speed of 20 mm/s with short irradiation time. At this scanning speed, open circuits were observed at the low pulse frequencies, indicating that the total power or total energy deposited into the polyimide surface was not sufficient for modifying it. As the scanning speed decreased to 12.5 mm/s, the rate of decrease of resistance with an increase in pulse frequency was much smaller and appeared to reach a limiting value at high frequency. This decrease of resistance with pulse frequency is to be expected as higher pulse frequency could increase the number of pulses deposited per laser spot and thus increasing the total power deposited per spot for the modification of the polyimide surface.

In contrast, and somewhat counter intuitively, at a moderately-high power of 18 W (power density of 12.40 W/cm^2^), even at a much higher scanning speed of 50 mm/s, the measured resistance value remained approximately constant and was independent of the pulse frequency; the resistance was also smaller as compared to the measured resistance at power of 5.0 W at the lower scanning speeds for frequencies investigated. This indicates that once the threshold of individual pulse energy for the modification of polyimide was reached, the surface modification, and thus the measured resistance, would be independent of pulse frequency and instead a function of the average laser power.

In summary, high electrical conductivity, namely low resistance, could be achieved at high power with high irradiation scanning speed independent of pulse frequency. If sufficiently high laser power cannot be delivered, conductivity may be achieved at low power with slow scanning speed with more than a single pass at high pulse frequency; however, this is not quite desirable as the value of the conductivity achieved would be lower and less consistent when it is a function of many parameters.

### 3.2. Morphology of the Irradiation Line

**Figure 6:**
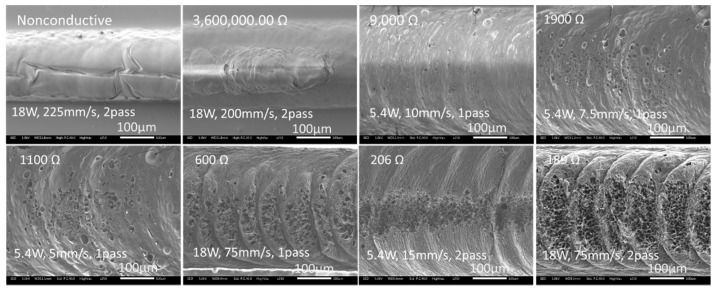
SEM images showing the polyimide morphology after laser irradiation (scanning from left to right in the images).

Figure 6 shows the morphology of the polyimide after irradiation at various laser parameter values resulting in different measured resistance. The nonconductive irradiation line had a smooth shrunk surface induced from laser melting solidification. With a decrease in electrical resistance, progressively the irradiated line showed a porous structure along the scanning line center. Indeed, the material modification was not uniform across the whole width of the scanning line, with much higher and extensive modification at the center of the line. The crescents appeared in the scanning belt indicated pulse overlapping, with each crescent corresponding to one pulse. These observations indicate that not the entire width of the irradiation line would be electrically conductive; highest conductivity would be at the line central portion where the most extensive modification was located. The observed material modification profile is to be expected as it corresponded to the Gaussian energy intensity profile of the laser beam, with modification most extensive at the center of the line where the laser energy intensity is the highest at the center of the beam.

**Figure 7:**
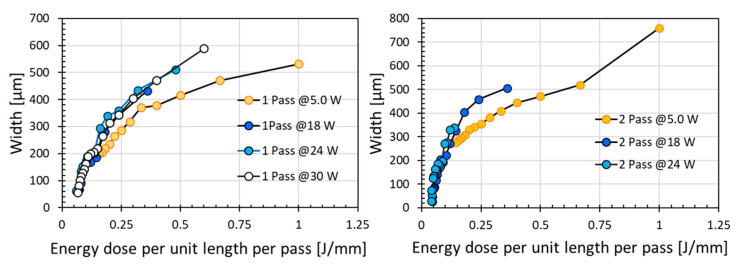
Scanning line width under various energy dose per unit length per pass for one or two passes (power of 5, 18, 24, and 30 W gave density of 3.44, 12.40, 16.54, and 20.67 W/cm^2^, respectively).

It is to be expected that with an increase in irradiation energy dose per unit length through an increase in laser power from 5 W to 30 W and a decrease in scanning speed from 450 mm/s to 5 mm/s, there was an obvious increase in irradiation line width as shown in Figure 7. This is because the laser–polyimide interaction was enhanced under a high energy dose per unit length, i.e., at high power with longer interaction time. Indeed, rather independent of the individual change in laser power or scanning speed, the line width produced under similar energy dose per unit length per pass would be similar, either scan one pass or two passes. At low energy dose per unit length at low laser power, only the tip of the gaussian beam had sufficient energy to induce modification of polyimide surface. Therefore, the line width was much less than one spot width (diameter) at this low energy dose. The line width would increase with an increase in energy dose per unit length and laser power. However, with sufficiently high laser power, this increase in line width would become less sensitive with an increase in laser power. Indeed, the critical role of laser power in addition to energy dose per unit length is highlighted by the relatively narrower line width at the lower laser power of 5.0 W despite of the same or higher energy dose per unit length as compared to that at higher laser power, see Figure 7.

**Figure 8:**
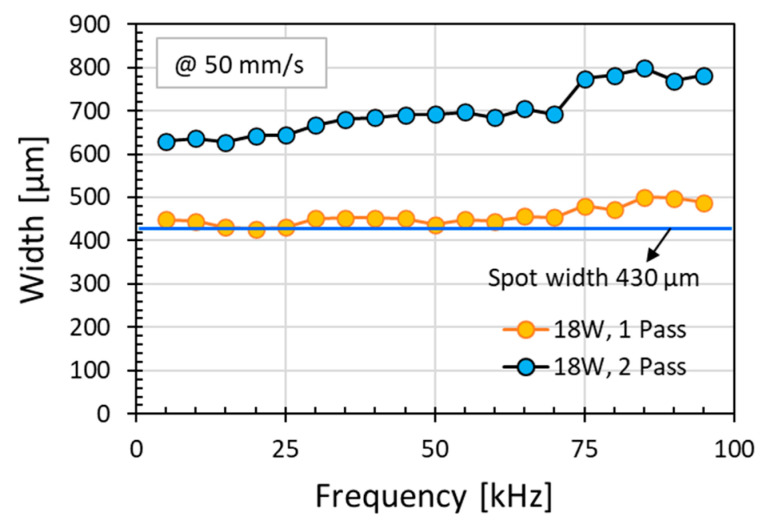
Scanning line width scanned under various laser pulse frequency with energy dose per unit length per pass of 0.36 J/mm for one or two passes obtained at power of 18 W (12.40 W/cm^2^) and speed of 50 mm/s. The laser spot width is indicated in the figure.

However, the changes in line width with frequency were not significant especially for single pass irradiation in Figure 8; this is consistent with the observed changes of resistance with frequency in Figure 5. When reaching the threshold of pulse energy to induce polyimide modification, the extent of modification would be a function of the average laser power and independent of pulse frequency. At high scanning speed of 225 mm/s, the polyimide surface was not sufficiently modified even at moderately high laser power of 18 W. As a result, not only the irradiation line width was narrow but also non-conductive.

For lines which had been modified conductively, the modification line width was significantly larger than the spot size of 430 µm in diameter, as observed in Figure 6. However, by comparing the morphology of conducting and non-conductive lines, the line width of the electrically conductive region was estimated to be around 220 µm, which was just about half of the laser spot diameter. This line width of the conductive region would be the key parameter for scanning line pitch with multiple overlapped line scanning for conductivity modification of an area.

**Figure 9:**
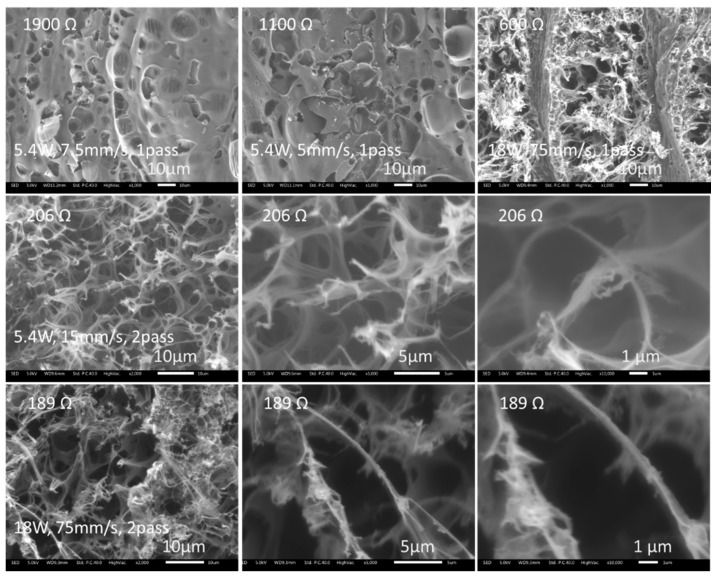
High magnification SEM images showing the porous structure transition to feather structure together with fiber structures with decreasing electrical resistance.

Figure 9 presents the detailed observation of the porous structure. Correlating these observations with that in Figure 6, it can be deduced that the more porous and extensive were the structure, the lower the electrical resistance measured. After laser irradiation, the examined morphology was modified from a smooth surface to bubble porous, feather porous, and or flank porous with nano level fiber structures with decreasing electrical resistance from 1900 Ω to 189 Ω. The low resistance morphology was typically multi-layered flakes or frameworks topography; this could well be the desired structure for high electrical conductivity induced under laser irradiation.

### 3.3. MicroRaman Analysis of Porous Structures

It has been previously identified that laser generation of a conductive layer is due to the local carbonization of polyimide under laser irradiation through photo-thermal and photo-degradation mechanisms [3,4,5,6]. For the photo-thermal mechanism, polyimide absorbs the incident photon energy and converts it into heat. This induces extremely high temperature in the irradiated region and results in carbonization [4]. For the photo-degradation mechanism, the transition of polyimide molecules from the ground state to the excited state after photon energy absorption results in chain breaks and the formation of free radicals. For a long-wavelength laser, the IR laser couples to the C-C bonds present in the carbon precursor and provides efficient photothermal heating [9]. The polyimide film absorbs the energy strongly to cause undesired effects, such as ablation, deformation, and even burning in air. It has been reported that the chain-growth polymers may undergo rapid depolymerization at the laser-induced temperatures. The mechanism of laser graphitization in polymers is strongly correlated to the structural features present in the repeat units, such as aromatic and imide repeat units. Polyimide contains aromatic and imide repeat units which can form graphene [6]. The detailed photochemical mechanisms responsible for the large change in electrical conductivity induced by laser radiation in polymers appear to be complex. To further elucidate these mechanisms, MicroRaman was employed to systematically investigate the modified region under irradiation with various laser parameters.

**Figure 10:**
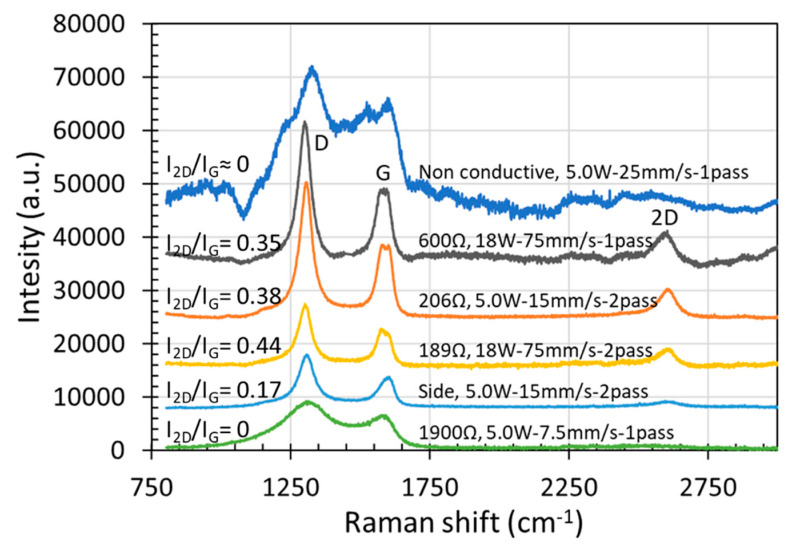
Raman spectra of the porous structures produced under various laser irradiation conditions.

Since the first measurement of the Raman spectrum of graphite [12], Raman scattering has become a popular characterization technique in carbons science and technology [13]. With a laser source of 785 nm, Figure 10 shows the Raman Spectra acquired from the various electrical resistance samples. Three peaks were clearly observed, namely D-peak at 1360 cm^−1^, G-peak at 1600 cm^−1^, and 2D-peak at 2700 cm^−1^; these peaks were identified to be graphene structures. Figure 10 agrees rather well with Wang et al. that at Raman laser excitation of 785 nm, G band was observed at 1581 cm^−1^ and 2D band at 2591 cm^−1^ [14].

The G-peak signifies the presence of carbon–carbon bond stretching, while the D-peak signifies the presence of disorder or impurities in the graphene sample which suggests the existence of defects in the graphene matrix [6,7]. The 2D to G ratio could provide a good indication of the existence of high-quality single layer graphene. The presence of high-quality single layer graphene may be confirmed when the ratio of I_2D_/I_G_ is greater than, or equal to 2 [15]. When the I_2D_/I_G_ ratio is below 0.6, it is generally accepted that the graphene film contains more than four layers, with a near certainty of more than five layers when I_2D_/I_G_ is below 0.4 [16]. From Figure 10, it can be deduced that no single layer graphene was synthesized under laser direct irradiation in air. The produced graphene was multiple layers, with the number of layers around 4–5. It further shows that the electrical resistance was a function of I_2D_/I_G_ ratio. Electrical resistance decreased with increasing I_2D_/I_G_ ratio. The non-conductive modification layer was observed to be at a I_2D_/I_G_ ratio of below 0.17 in Figure 10, indicating the many layer-structure (>5) of graphene.

In view of the graphene intrinsic electric property, it has been reported that the electrical conductivity was related to the graphene layers. Higher electrical conductivity is associated with less layers of graphene structures [17]. The higher the ratio of I_2D_/I_G_, the smaller the number of graphene layers, and the higher the electrical conductivity. However, the electrical resistance of a material not only depends on its intrinsic electric property but also relies on the dimensions of its physical structure. Correlating the observations here to the irradiation line width (Figure 7 and Figure 8) and the graphene porous structure (Figure 6), obviously, lower resistance was achieved at wider irradiation line width of the multi-layered graphene porous structures. The measured resistance values over the irradiated 10 mm length in polyimide surface were a convolution results of multiple factors, namely the multiple layered graphene structure and the irradiation cross-section area of the graphene structures (i.e., the modification thickness and width). Everything being equal, the larger the irradiation region, the lower would be the electrical resistance.

Furthermore, the prominent peak of D and G bands as well as their large intensity ratios in Figure 10 would indicate a certain amount of defects, probably oxidation induced in air environment, which could be related to the unclear edges and large pores observed in the SEM images in Figure 9. The porous graphene frameworks consisted of interconnected multi-layered graphene sheets, a typical feature of the randomly stacked multi-layered graphene which corresponds to the symmetric profile of the 2D band, with full width at half maximum of ∼50 cm^−1^ in Figure 10. The 2D peak position upshifts to 50 cm^−1^ when the number of layers increases from 1 to 5 [16]. It has been revealed that electrical conductivity of graphene derivatives strongly depends on the oxidation level [18]. Therefore, to achieve a single layered graphene structure with good electrical conductivity, probably, laser irradiation under vacuum, inert gas or even chemically active environment would be preferred for microelectronic device manufacturing.

## 4. Conclusions

Graphene structures were produced on polyimide surface through a single scan of CO_2_ laser beam under air atmosphere. SEM characterization revealed that the graphene had a morphology of thin wall porous structures together with nano level fiber structures. The electrical resistance of the laser irradiation line was dominated by the graphene structures generated at the irradiation line center instead of uniformly across the whole line width; the dominant electrically conductive center line width was about half of the laser spot diameter. This conductive width would be a key indicator for the line pitch necessary for multiple overlapped scanning lines for achieving a conductive area on a polyimide surface. Micro-Raman characterization found that for the electrically conductive layer produced on the polyimide surface, the graphene had a multiple layer structure of 4–5 layers indicated by a I_2D_/I_G_ ratio of 0.3–0.5. A laser parametric study concluded that to achieve high electrical conductivity effectively required sufficiently high laser power for the modification of polyimide surface. From a practical application perspective, a single scan with sufficiently high laser power at a high speed is preferred than multiple scans with low laser power at a slow speed. With sufficiently high laser power, the electrical conductivity after laser surface modification was a function of laser average power and was independent of pulse frequency; the resistance reduction was dominantly a function of the energy dose per unit length. However, with a low and therefore insufficiently high laser power, resistance reduction was complicated and was a function of both the energy dose per unit length and the pulse frequency. At a constant energy dose per unit length at low laser power, the interval time between pulses became important in determining the resistance reduction. Resistance reduction was more effective at a shorter pulse interval time with an increase in pulse frequency. More importantly, low laser power was not effective in enhancing electrical conductivity, even with multiple passes. In addition, the trend of resistance reduction was consistent with the modified line width and pulse spot overlapping observed in irradiation morphology. With sufficiently high laser power, the increase in line width was insensitive with laser power and was a function of energy dose per unit length. At low laser power, obvious increase in line width was a result of an increase in energy dose per unit length through the increase in laser power and decrease in scanning speed. The electrical resistance decreased from a few hundred Ohms to 30 Ohms when the energy dose per unit length increased from 0.16 J/mm to 1.0 J/mm, i.e., laser power increased from 5.0 W to 24 W (power density of 3.44 × 10 W/cm^2^ to 16.54 W/cm^2^ respectively) at a scanning speed of 12.5 mm/s. In contrast, for laser power below 5 W at speeds exceeding 22.5 mm/s resulted in non-conductive open loop.
